# Operational Factors Associated With National Guideline Adherence to Vaso-Occlusive Crisis Management in the Emergency Department

**DOI:** 10.1016/j.acepjo.2026.100475

**Published:** 2026-07-28

**Authors:** Melinda R. Rushing, Mafudia A. Suaray, Alice J. Cohen, Robert Eisenstein, Ethan A. Halm, Shawna V. Hudson

**Affiliations:** 1 Rutgers University, Edward J Bloustein School of Planning and Public Policy, New Brunswick, New Jersey, USA; 2 Department of Family Medicine and Community Health, Rutgers Robert Wood Johnson Medical School, New Brunswick, New Jersey, USA; 3 Robert Wood Johnson Barnabas Health, Newark Beth Israel Medical Center, Newark, New Jersey, USA; 4 Department of Emergency Medicine, Rutgers Robert Wood Johnson Medical School, New Brunswick, New Jersey, USA; 5 Department of Medicine, Rutgers Robert Wood Johnson Medical School, New Brunswick, New Jersey, USA

**Keywords:** sickle cell disease, guideline adherence, pain management, vaso-occlusive crisis

## Abstract

**Objectives:**

The National Heart, Lung, and Blood Institute guidelines for sickle cell disease (SCD) vaso-occlusive crisis (VOC) management recommend emergency departments (EDs) rapidly assess pain and administer pain medication following an individual’s arrival. An ED’s experience treating SCD along with co-occurring operational factors may affect guideline adherence. We investigated the effect of operational factors on VOC guideline adherence within a large New Jersey health system.

**Methods:**

Electronic health record data between January 2022 and September 2025 were analyzed. Outcomes: rate of guideline-concordance, time-to-first and time-to-second pain medication administration. Adherence was defined as first dose ≤30 minutes after triage and second dose *≤*30 minutes after first. Predictors: SCD patient volume, ED volume, shift, and emergency severity index (ESI) score. Descriptive and mixed-effects regression analyses were conducted.

**Results:**

There were 7160 ED visits by 1401 patients. 14.2% of encounters were adherent for first pain medication, and 52.6% for second. SCD volume, ED volume, shift, and ESI score were associated with guideline-concordant encounter rates, but not site-level decreased average times. Shift timing was the only exposure significantly associated with mean times to both first and second pain medication administration. Individuals arriving during the evening shift received their first pain medication 15.93 minutes later (95% CI: 2.43 to 29.37) and their second pain medication 10.92 minutes later (95% CI: 4.01 to 17.83) than those arriving during the night shift.

**Conclusion:**

Several operational factors were associated with the likelihood of guideline-concordant encounters; whereas, only shift timing influenced mean times to pain medication administration, highlighting a distinction between guideline concordance and overall timeliness.

**The Bottom Line**

We examined how the emergency department operates affects care for people with sickle cell disease during pain crises. Using electronic health record data from 2022 to 2025, we looked at how patient volumes, overall emergency department activity, time of day, and triage severity influenced care. We found these factors were linked to whether patients received care that followed national guidelines, but they did not improve how quickly pain medication was given overall. Only the time of day (shift) was consistently tied to faster treatment. These findings suggest improving guideline-based care and reducing delays in pain treatment may require different strategies.

## 1 Introduction

### 1.1 Background

In 2014, the National Heart, Lung, and Blood Institute (NHLBI) published evidence-based national guidelines for managing sickle cell disease (SCD). The guidelines recommend emergency departments (EDs) rapidly assess and quickly administer pain medication following the arrival of an individual with SCD for treatment for an acute pain episode, ie, vaso-occlusive crisis (VOC).^1^ The guidelines recommend the first dose of pain medication be administered within 30 minutes of triage and the second 30 minutes after the first.^1^ However, adherence to timely pain medication administration in real world practice is challenging.^2^^,^^3^

More than 10 years after these guidelines were published, individuals with SCD continue to experience delays in receiving initial and subsequent doses of pain medication when they arrive at the ED, resulting in prolonged suffering.^2^^,^^4^^,^^5^ In addition to the complexities inherent to clinical decision-making, operational factors, such as ED crowding, variations in emergency severity index (ESI) scoring, different prioritization across age groups, and clinical experience treating the disease continue to impede timely pain management for this population.^4^^,^^6^, ^7^, ^8^, ^9^

### 1.2 Importance

A key determinant of effective SCD management is providers’ ability to promptly recognize SCD-related complications and initiate treatment, often reflective of clinicians’ experience treating the disease.^3^^,^^10^ Providers who have experience managing SCD report greater confidence in treating SCD complications, which may translate to better guideline adherence.^11^^,^^12^ However, it remains unclear how experience extends to ED system-level performance—do institutions that treat larger SCD volumes have better guideline adherence? When analyzed independently, sites with higher SCD volumes did not have overall better times to first pain medication administration than lower-volume sites, but the rate of adherent encounters was not investigated.^13^ While this reveals an overall trend, it does not illuminate the system-level factors that contribute to when guideline timeframes are met vs missed.

### 1.3 Goals of This Investigation

Building on prior work, this analysis examines the association between co-occurring ED operational factors—including SCD volume, overall ED volume, ESI assignment, and time of arrival—and NHLBI guideline adherence. By evaluating these factors within a single health system, our study aimed to better understand how operational variation influenced timeliness of pain management beyond experience alone. This study investigated: (1) the associations between operational factors and rates of guideline-concordant encounters; and (2) the effect operational factors have on time to pain medication administration. Investigating these associations will help identify areas amenable to intervention.

## 2 Methods

### 2.1 Study Design

We conducted a retrospective observational study using data extracted from the Epic electronic health record for 13 EDs in the Robert Wood Johnson Barnabas Health care system (RWJBarnabas). The study population included all RWJBarnabas encounters, with the sample limited to SCD-related encounters. We applied Snyder et al^14^ definition for SCD encounters using ICD-10 codes D57, D57.0X, D57.1, D57.2X, D57.4X, and D57.8X. All ED encounters with a primary or secondary SCD ICD-10 code between January 2022 and September 2025 were examined. Each ED treated at least one individual with SCD during the study period and was either an academic, teaching, or community hospital. Data were analyzed at the encounter level.

### 2.2 Outcomes

Our primary outcome was the rate of guideline adherence, and our secondary outcomes were time-to-first and time-to-second pain medication administration. Guideline concordance was defined as administration of both the first and second pain medications within 30 minutes. Time-to-first medication was measured from triage to the first dose, and time-to-second medication from the first to the second dose.

### 2.3 Exposures

Our main exposures were SCD patient volume (high: 250+ encounters/y vs low: <250 encounters/y), overall ED volume (high: 50,000+ encounters/y vs low: <50,000 encounters/y), adherence to recommended ESI score (adherent: <3 vs nonadherent: 3+), and shift when arrived (Day = 7 am–2:59 pm, Evening = 3 pm to 10:59 pm, Night = 11 pm–6:59 am). The total number of SCD encounters for the system was divided into tertiles, with the high-volume category defined as the third tertile (hospitals with 250+ SCD encounters/y). We adjusted our models for age (pediatric = 0-17, young adult = 18-30, adult = 31+), and biological sex.

### 2.4 Data Analysis

First, counts, percentages, medians, and chi-squared statistics were calculated in a bivariate analysis. Then, we utilized mixed-effects regression modeling with cluster-robust standard errors to assess the effect operational factors had on times to first and second pain medication administration. The mixed-effects model accounted for clustering at the ED site level through a random site effect, whereas cluster-robust standard errors were used to address within-patient clustering.^15^ Analyses were conducted using SAS 9.4. The study was approved by the Rutgers University institutional review board.

## 3 Results

There were 7160 ED encounters among 1401 unique patients where 82.3% were Black, 54.7% were female and 43.5% were 31+ years old. The median time-to-first pain medication was 73 minutes (Interquartile range (IQR): 43 to 117), and 28 minutes for the second (IQR: 2-80) (Table 1). Guideline adherence rates were 14.2% for first pain medication and 52.6% for second. Four EDs were classified as high SCD volume sites, with SCD patient volumes ranging from 129-441 across 45 months, while low-volume sites ranged from 5-91. Six EDs were classified as high ED volume ranging from 56,126 to 104,836 all-cause encounters annually, while the low-volume sites ranged from 14,488-45,437 encounters annually. The bivariate analysis revealed SCD volume was associated with guideline adherence for first (*P* = .04) and second (*p* = .002) pain medication administration (Table 1). Among the high SCD volume sites, 14.7% of encounters were guideline adherent for first-dose (n = 828) and 51.6% for second-dose (n = 2786). Overall ED volume was not associated with guideline adherence for first pain medication administration (*p* = .95), but it was for the second (*p* = .03). The night shift had the highest proportion of guideline-adherent encounters for both first and second pain medication administration compared to evening and day shifts; however, this association was statistically significant only for the first dose (16.6% vs 13.0% and 13.7%, *P* = .002). There was a significant association between ESI score adherence and guideline adherence to time-to-first pain medication dose (*P* < .001), but not for the second dose (*P* = .73). Pediatric encounters had the highest proportion of guideline-adherent first pain medication administration (29.3%, *P* < .001), whereas adult encounters had the highest proportion of adherent second pain medication administration (55.9%, *P* < .001). There was a significant association between biological sex and rate of guideline adherence to time-to-first pain medication dose (*P* < .001), but not for the second dose (*P* = .11).

**Table 1:** Descriptive analysis of operational factors and association with adherence to first and second pain medication to treat individuals with sickle cell disease at the encounter level.

Variables	Adherent to 1st pain medication dose N (Row%)	Nonadherent to 1st pain medication dose N (Row%)	∗*P* value	Adherent to 2nd pain medication dose N (Row%)	Nonadherent to 2nd pain medication dose N (Row%)	∗*P* value
	1019 (14.2)	6141 (85.8)		3572 (52.6)	3223 (47.4)	
SCD visit volume						
High	828 (14.7)	4816 (85.3)	.04	2786 (51.6)	2610 (48.4)	.002
Low	191 (12.6)	1325 (87.4)		786 (56.2)	613 (43.8)	
ED volume			.95			.03
High	854 (14.2)	5142 (85.8)		2977 (52)	2746 (48)	
Low	165 (14.2)	999 (85.8)		595 (55.5)	477 (44.5)	
Shift						
Day	375 (13.7)	2369 (86.3)	.002	1366 (52.6)	1230 (47.4)	.75
Evening	320 (13)	2139 (87)		1211 (52)	1117 (48)	
Night	324 (16.6)	1633 (83.4)		995 (53.2)	876 (46.8)	
ESI score			<.001			.73
Adherent	402 (12.1)	2924 (87.9)		1644 (52.8)	1470 (47.2)	
Nonadherent	617 (16.1)	3217 (83.9)		1928 (52.4)	1753 (47.6)	
Age						
0-17 y	327 (29.3)	788 (70.7)	<.001	351 (36.2)	619 (63.8)	<.001
18-30 y	391 (13.5)	2513 (86.5)		1529 (54.7)	1267 (45.3)	
31+ y	301 (9.6)	2840 (90.4)		1692 (55.9)	1337 (44.1)	
Sex			<.001			.11
Female	502 (12.1)	3629 (87.8)		2115 (53.4)	1847 (46.6)	
Male	517 (17.1)	2512 (82.9)		1457 (51.4)	1376 (48.6)	
	Median Minutes to First Pain Medication Dose for all Encounters Min (IQR)			Median Minutes to Second Pain Medication Dose for all Encounters Min (IQR)		
	73 (43-117)			28 (2-80)		

SCD, sickle cell disease.

∗ *P* values for the results of the chi-squared test conducted for the bivariate analyses.

When time-to-first pain medication was examined, no significant differences were observed across SCD volume, overall ED volume, or ESI score groups after adjustment for age and sex (Table 2). In contrast, individuals arriving during the evening shift received their first pain medication an average of 15.93 minutes later (95% CI: 2.43 to 29.37) than those arriving during the night shift. No significant difference in time-to-first pain medication was observed between the day shift and the night shift after adjustment for age and sex.

**Table 2:** Results of the mixed-effects regression models with cluster-robust standard errors to analyze the effect operational factors had on time to pain medication administration.

Predictor	Different in time	95% CI	
Time-to-first pain medication			
High SCD volume vs low SCD volume	7.16	−12.36	26.68
High ED volume vs low ED volume	5.67	−17.46	28.81
Day vs night	5.73	−1.24	12.72
**Evening vs night**	15.93	2.52	29.35
ESI score nonadherent vs ESI score adherent	−4.57	−16.87	7.72
**18-30 years oldvs 0-17 years old**	26.95	18.25	35.65
**31+ years oldvs 0-17 years old**	33.59	26.19	40.99
**Male vs female**	−8.05	−14.22	-1.88

Time-to-second pain medication			
High SCD volume vs low SCD volume	6.92	−15.95	29.79
High ED volume vs low ED volume	−7.05	−31.84	17.73
Day vs night	9.93	−0.43	20.31
**Evening vs night**	10.92	4.01	17.83
ESI score nonadherent vs ESI score adherent	14.00	−4.07	32.07
**18-30 years oldvs 0-17 years old**	−64.30	−84.19	−44.41
**31+ years oldvs 0-17 years old**	−54.70	−75.28	−34.12
**Malevs female**	8.42	2.13	14.72

ED, emergency department; SCD, sickle cell disease.

When time-to-second pain medication was assessed, a similar pattern was observed after adjustment for age and sex (Table 2). No significant differences were observed across SCD volume, overall ED volume, or ESI score groups. However, individuals arriving during the evening shift received their second pain medication an average of 10.92 minutes later (95%W CI: 4.01 to 17.83) than those arriving during the night shift after adjustment for age and sex.

## 4 Limitations

First, generalizability of our results to other settings is uncertain, although the EDs evaluated represent a mix of academic medical centers, community teaching hospitals, and nonteaching hospitals; they are not all inclusive of facility type. Second, our sample size at each ED may have limited power to detect modest associations. We aimed to address this issue by using cluster-robust regression modeling. Third, we were unable to assess key operational factors—such as bed occupancy, length of stay, patients leaving without being seen, order set use, and medication route—that may better explain guideline nonadherence. Finally, we were unable to control for important patient-level variables, such as SCD severity, comorbidities, or operational factors, such as clinical decision support utilization.

## 5 Discussion

Our findings distinguish adherence from timeliness: operational factors increased guideline-concordant care but did not consistently reduce time to medication. We expected increased system-level experience managing SCD would lead to much shorter wait times for pain medication administration; however, our findings suggest there are other factors that need to be considered beyond experience when it comes to timely medication administration. Average times to first and second doses did not differ significantly by SCD volume, likely reflecting similar rates of guideline adherence across sites (14.7% at high-volume vs 12.6% at low-volume EDs). Although high SCD-volume sites had a greater proportion of guideline-concordant encounters, this difference was not sufficient to reduce overall average times to pain medication. These findings suggest that experience managing SCD alone is insufficient to improve system-level timeliness, underscoring the need for further investigation into the factors driving persistent delays.

During the study period, encounters were concentrated in two ED, likely reflecting higher overall patient volumes driven by local community demographics. Despite greater experience managing SCD, these sites continued to struggle with consistent guideline adherence, reinforcing prior findings that experience alone does not eliminate delays in pain management.^16^ Because ED crowding has been shown to affect SCD care, we used overall ED volume as a proxy measure. Guideline adherence for the first pain medication dose did not differ between high- and low-volume EDs, whereas adherence for the second dose was slightly higher at lower-volume sites (55.5% vs 52%). These findings suggest a modest difference in adherence for second pain medication dosing by ED volume, which may reflect the influence of crowding and warrants further investigation. Additionally, guideline adherence for both the first and second pain medication doses was higher during the night shift, and night shift average times to both doses were shorter. Emergency departments typically have lower patient census during the morning and late-night shifts, resulting in shorter wait times.^17^ This pattern suggests reduced ED crowding during the night shift may facilitate more timely pain medication administration for individuals with SCD. However, this does not represent a feasible solution, as patients cannot be expected to seek care exclusively at night. Because ED crowding is not readily modifiable, identifying additional workflow factors beyond nighttime conditions is needed.

Adherence to ESI scoring was associated with guideline concordance for first pain medication administration, but not for second. NHLBI guidelines recommend assigning an ESI score of 2 or lower for a VOC to ensure individuals with SCD are prioritized, as ESI score significantly influences wait times for SCD treatment.^1^^,^^7^ Our findings suggest ESI adherence primarily influences initiation of pain management, though not in the expected direction: nonadherent encounters had higher rates of guideline-adherent first-dose administration compared with adherent encounters (16.1% vs 12.1%). This does not suggest higher ESI scores are preferable but highlights the ED workflow importance. Some RWJBarnabas EDs utilize rapid treatment areas, allowing patients triaged at a higher ESI to be seen more quickly. In these settings, patients with SCD who were assigned higher ESI scores may have moved through the ED workflow more efficiently, increasing the likelihood of timely pain medication administration at certain sites. This hypothesis warrants further investigation, as ESI implementation and associated workflows may inadvertently facilitate guideline-adherent care.

Across the system, 52.6% of all encounters met guidelines for time-to-second pain medication vs 14.2% for the first, suggesting there is a greater barrier to initiating treatment for VOCs than maintaining treatment for VOCs. Our findings identify a potential leverage point within the system: reducing barriers to initiating VOC treatment may improve guideline-concordant administration of both first and second pain medication doses, ultimately reducing suffering for individuals with SCD. Further investigation is needed to identify the barriers to first pain medication dose.

Several operational factors were associated with the likelihood of guideline-concordant encounters, whereas only shift timing influenced mean times to pain medication administration, highlighting a distinction between guideline concordance and overall timeliness. Greater adherence for second-dose compared with first-dose administration suggests a potential leverage point for improvement, and the unexpected association with ESI score underscores the role of ED workflow. Further work is needed to identify modifiable targets to improve timely pain management for individuals with SCD.

## Author Contributions

MRR led the design, analysis, and manuscript drafting for this study. MAS, AJC, and RE assisted in interpreting the results and edited the manuscript. LZ contributed to the development of the manuscript. EAH, and SVH advised on the design of the study, interpreting the results, and edited the manuscript.

## Funding and Support

This study was funded by AHRQ Learning Health System Scholar (P30 HS029759).

## Data Sharing Statement

The entire deidentified data set, data dictionary, and analytic code for this investigation are available on request, from the date of article publication, by contacting Rutgers University Clinical and Research Data Warehouse team at crdw_requests@oarc.rutgers.edu.

## Conflict of Interest

All authors have affirmed they have no conflicts of interest to declare.

All authors attest to meeting the four ICMJE.org authorship criteria: (1) Substantial contributions to the conception or design of the work; or the acquisition, analysis, or interpretation of data for the work; AND (2) Drafting the work or revising it critically for important intellectual content; AND (3) Final approval of the version to be published; AND (4) Agreement to be accountable for all aspects of the work in ensuring that questions related to the accuracy or integrity of any part of the work are appropriately investigated and resolved.
